# Glycosylation of Ganoderic Acid G by *Bacillus* Glycosyltransferases

**DOI:** 10.3390/ijms22189744

**Published:** 2021-09-09

**Authors:** Jiumn-Yih Wu, Hsiou-Yu Ding, Tzi-Yuan Wang, Yun-Rong Zhang, Te-Sheng Chang

**Affiliations:** 1 Department of Food Science, National Quemoy University, Kinmen County 892, Taiwan; wujy@nqu.edu.tw; 2 Department of Cosmetic Science, Chia Nan University of Pharmacy and Science, No. 60, Erh-Jen Rd., Sec. 1, Jen-Te District, Tainan 71710, Taiwan; ding8896@gmail.com; 3 Biodiversity Research Center, Academia Sinica, Taipei 11529, Taiwan; tziyuan@gmail.com; 4 Department of Biological Sciences and Technology, National University of Tainan, Tainan 70005, Taiwan; S10758011@gm2.nutn.edu.tw

**Keywords:** *Bacillus*, *Ganoderma* *lucidum*, glycosyltransferase, saponin, triterpenoid

## Abstract

*Ganoderma lucidum* is a medicinal fungus abundant in triterpenoids, its primary bioactive components. Although numerous *Ganoderma* triterpenoids have already been identified, rare *Ganoderma* triterpenoid saponins were recently discovered. To create novel *Ganoderma* saponins, ganoderic acid G (GAG) was selected for biotransformation using four *Bacillus* glycosyltransferases (GTs) including BtGT_16345 from the *Bacillus thuringiensis* GA A07 strain and three GTs (BsGT110, BsUGT398, and BsUGT489) from the *Bacillus subtilis* ATCC 6633 strain. The results showed that BsUGT489 catalyzed the glycosylation of GAG to GAG-3-*o*-*β*-glucoside, while BsGT110 catalyzed the glycosylation of GAG to GAG-26-*o*-*β*-glucoside, which showed 54-fold and 97-fold greater aqueous solubility than that of GAG, respectively. To our knowledge, these two GAG saponins are new compounds. The glycosylation specificity of the four *Bacillus* GTs highlights the possibility of novel *Ganoderma* triterpenoid saponin production in the future.

## 1. Introduction

*Ganoderma lucidum* is a medicinal fungus that has been used for health improvement and the treatment of various diseases in Asia over thousands of years [1]. In modern studies, both triterpenoids and polysaccharides have been demonstrated to exhibit bioactivity [2]. Triterpenoids contain six isoprene units and usually form tetra- or penta-cyclic structures. Triterpenoids are widely noted to exist in plants and some fungi. Until now, more than 14,000 different triterpenoids have been identified, and many of them have been proven to play important roles in bioactivity [3]. Triterpenoid saponins are important natural products in plants, and some triterpenoid saponins exhibit greater bioactivity than triterpenoid aglycones [4]. Therefore, the glycosylation of triterpenoids is important for the biosynthesis of triterpenoid saponins. *G. lucidum* also contains large amounts of triterpenoids, and over 300 triterpenoids that form *Ganoderma* sp. have been identified previously [5,6,7]. All *Ganoderma* triterpenoids belong to the tetracyclic lanostane type of triterpenoids which contain different types of main skeletons, including ganoderic acids. These *Ganoderma* triterpenoids were found to possess varying degrees of bioactivity. For example, a *Ganoderma* triterpenoid, ganoderic acid A (GAA), was recently shown to have bioactive medical applications including the inhibition of bleomycin-induced lung fibrosis in mice [8], the attenuation of lipopolysaccharide-induced lung injury in mice [9], the retardation of renal cyst development in polycystic kidney disease [10], and the protection of neural cells against NO stress injury [11]. Considering the diversity of the *Ganoderma* triterpenoid group, studying the bioactivity of *Ganoderma* triterpenoids has become a core focus of biotechnology studies in recent years. Consequently, rare *Ganoderma* triterpenoid saponins have been identified recently [5,6,7].

**Figure 1:**
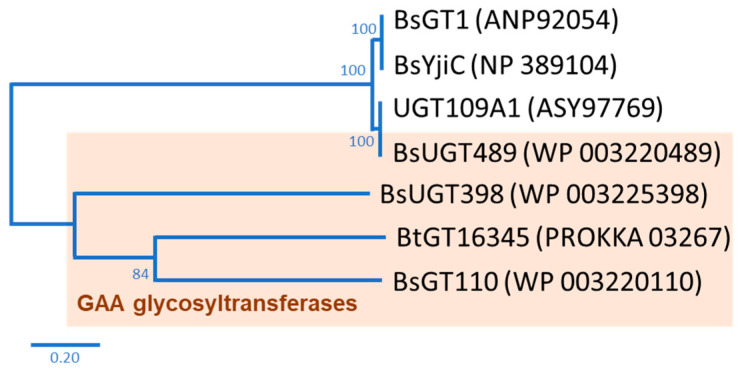
Seven known bacterial glycosyltransferases (GTs) from previous studies. The phylogenetic tree was adopted from Figure 1 in the study by Chang et al. [14].

In nature, the glycosylation of triterpenoids is initiated by glycosyltransferase (EC 2.4.1.X; GT) [12]. GT uses a nucleotide-activated sugar donor, such as uridine diphosphate glucose (UDP-G), to transfer the sugar moiety to a sugar acceptor. According to the carbohydrate-activating enzyme database there are 117 subfamilies of GTs, with over 850,000 GTs having already been identified [13]. However, only some rare bacterial GTs are able to glycosylate triterpenoids to triterpenoid saponins [14]. In our previous studies, we used GAA as a precursor to screen microbes with the ability to biotransform GAA to GAA saponins. Two bacteria, the *Bacillus subtilis* (Bs) American Type Culture Collection (ATCC) 6633 strain and the *Bacillus thuringiensis* (Bt) GA A07 strain, were able to produce GAA saponins. Four *Bacillus* GTs were further identified, based on their genomes, as the major enzymes able to catalyze the glycosylation of GAA to GAA saponins. BsGT110 [15], BsUGT398, and BsUGT489 [16] were identified using the ATCC 6633 strain, and BtGT_16345 was identified using the GA A07 strain [17]. So far, only seven *Bacillus* GTs with triterpenoid glycosylation activity have been identified, including the four *Bacillus* GTs mentioned previously [14]. Phylogenetic analysis suggests that the four *Bacillus* GTs are genetically distinct from each other (Figure 1) [14]. Although the four *Bacillus* GTs have been proven to have the ability to produce *Ganoderma* triterpenoid saponins, previous studies have only focused on GAA glycosylation [15,16,17]. Thus, it is worth investigating the catalytic activity of the four *Bacillus* GTs toward other triterpenoids.

Therefore, the glycosylation sites of ganoderic acid G (GAG), namely, the C-3, C-7, and C-12 hydroxyl groups and the C-26 carboxyl group, were selected to test the biotransformation of the four *Bacillus* GTs. The glycosylated saponins produced by the four *Bacillus* GTs using GAG were purified and their chemical structures were identified. In addition, the aqueous solubility of the GAG saponins was determined and compared with that of GAG. Accordingly, the catalytic specificity of the four *Bacillus* GTs toward the different functional groups of the ganoderic acids was clarified.

## 2. Results and Discussion

### 2.1. Biotransformation

**Figure 2:**
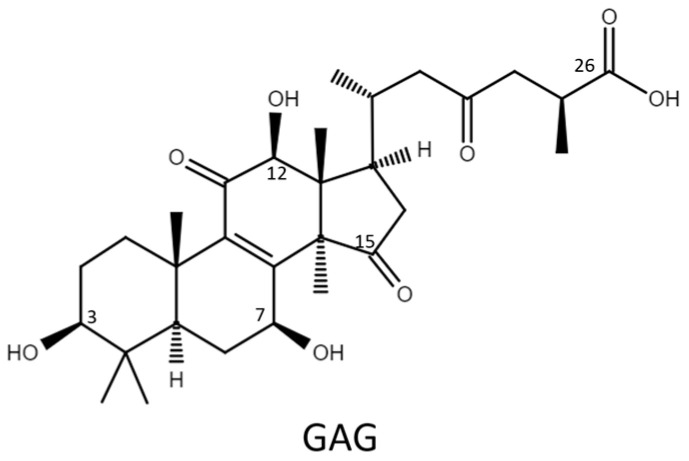
The chemical structure of ganoderic acid G (GAG).

**Figure 3:**
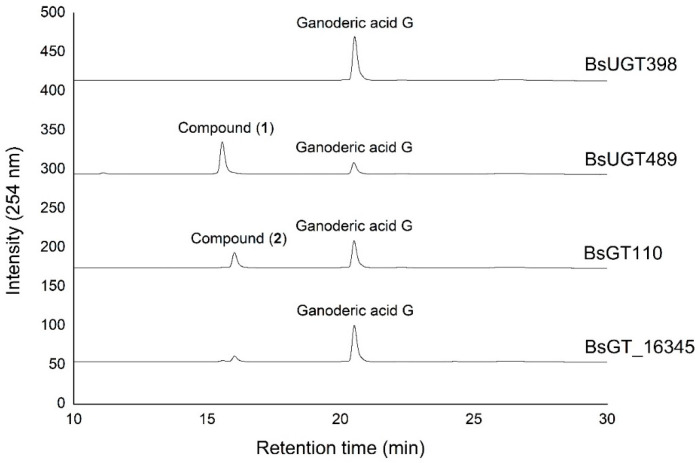
High-performance liquid chromatography (HPLC) results of the biotransformation products of GAG using the four *Bacillus* GT enzymes. The biotransformation and HPLC conditions are described in the Materials and Methods section.

Ganoderic acid is one of most common types of *Ganoderma* triterpenoids and primarily contains five functional groups that are available for modification, including the C-3, C-7, C-12, and C-15 hydroxyl groups and the C-26 carboxyl group [5,6,7]. In this study, the four *Bacillus* GTs (Figure 1) were assayed for their glycosylation activity toward GAG containing C-3, C-7, and C-12 hydroxyl groups and the C-26 carboxyl group (Figure 2). After the completion of the biotransformation reaction, the reaction mixture was analyzed using high-performance liquid chromatography (HPLC), the results of which are shown in Figure 3. The results indicate that BsUGT489 and BsGT110 biotransformed GAG to compound (**1**) and compound (**2**), with yields of 74% and 35%, respectively. Both BsUGT398 and BtGT_16345 showed little to no activity toward GAG, respectively. 

### 2.2. Purification and Identification of Biotransformed Products

**Figure 4:**
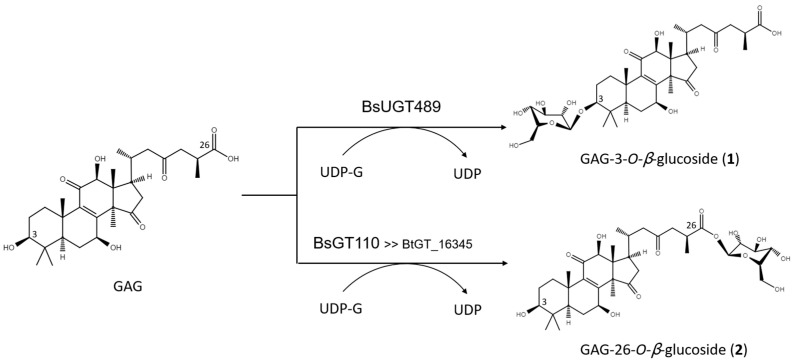
The biotransformation process of GAG to GAG saponins by the *Bacillus* GTs.

The biotransformation reactions of GAG with BsUGT489 and BsGT110 were selected to purify compound (**1**) and compound (**2**), respectively. The two biotransformation reactions were scaled up to 20 mL and the products were purified by preparative HPLC. From the 20 mL reaction mixture, 14.4 mg of compound (**1**) and 7.8 mg of compound (**2**) were purified. The molecular weights of the purified products were then determined by mass spectrometry. The mass spectrometer showed an [M−H]^−^ ion peak at m/z: 693.5 in the electrospray ionization mass spectrum (ESI-MS), corresponding to the molecular formula C_36_H_54_O_13_. The mass data imply that both compound (**1**) and compound (**2**) contain one glucosyl moiety attached to the GAG structure (Figures S1 and S2). To identify the structures in advance, the structures of both products were determined using nuclear magnetic resonance (NMR) spectroscopy. The ^1^H and ^13^C NMR, including the distortionless enhancement by polarization transfer (DEPT), heteronuclear multiple bond connectivity (HMBC), heteronuclear single quantum coherence (HSQC), nuclear Overhauser effect spectroscopy (NOESY), and correlation spectroscopy (COSY) spectra were obtained. The NMR spectra of compound (**1**) exhibited characteristic glucosyl signals, with the anomeric proton signal at *δ*_H_ 3.98 (^1^H, ddd, *J* = 8.6, 5.6, 2.1 Hz, H-5′), 4.03 (^1^H, t, *J* = 8.6 Hz, H-2′), 4.22 (^1^H, t, *J* = 8.6 Hz, H-4′), 4.24 (^1^H, t, *J* = 8.6 Hz, H-3′), 4.40 (^1^H, dd, *J* = 11.9, 5.6 Hz, H-6′a), 4.57 (^1^H, dd, *J* = 11.9, 2.1 Hz, H-6′b), and 4.91 (^1^H, d, *J* = 8.6 Hz, H-1′); and the anomeric carbon signal at *δ*_C_ 63.0 (C-6′), 71.8 (C-4′), 75.7 (C-2′), 78.4 (C-5′), 78.7 (C-3′), and 107.0 (C-1′). The large coupling constant (8.6 Hz) of the anomeric proton H-1′ (4.91 ppm) indicated the *β*-configuration. An ether linkage between the H-1′ of glucose and C-3 (4.91/88.3 ppm) of GAG was proven by the HMBC and NOESY (H-3/H-1′) spectra. The structure of compound (**1**) was thus confirmed to be GAG-3-*o*-*β*-glucoside. The NMR spectroscopic data are shown in Figures S3–S9. The signals of compound (**2**) were attributed to a glucose moiety, with *δ*_H_ 4.03 (^1^H, ddd, *J* = 8.8, 4.9, 2.8 Hz, H-5′), 4.20 (^1^H, t, *J* = 8.8 Hz, H-2′), 4.29 (^1^H, t, *J* = 8.8 Hz, H-3′), 4.35 (1H, t, *J* = 8.8 Hz, H-4′), 4.36 (^1^H, dd, *J* = 11.9, 4.9 Hz, H-6′a), 4.46 (^1^H, dd, *J* = 11.9, 2.8 Hz, H-6′b), and 6.33 (^1^H, d, *J* = 8.8 Hz, H-1′); and *δ*_C_ 62.1 (C-6′), 71.0 (C-4′), 74.2 (C-2′), 78.5 (C-3′), 79.5 (C-5′), and 96.3 (C-1′). The cross peak of H-1′ with C-26 (6.33/175.0 ppm) in the HMBC spectrum demonstrated the structure of compound (**2**) to be GAG-26-*o*-*β*-glucoside. The NMR spectroscopic data are shown in Figures S10–S16. The structures of the GAG saponins and the biotransformation process are shown in Figure 4. 

### 2.3. Catalytic Specificity of the Bacillus GTs for the Glycosylation of Functional Groups on Ganoderic Acids

**Figure 5:**
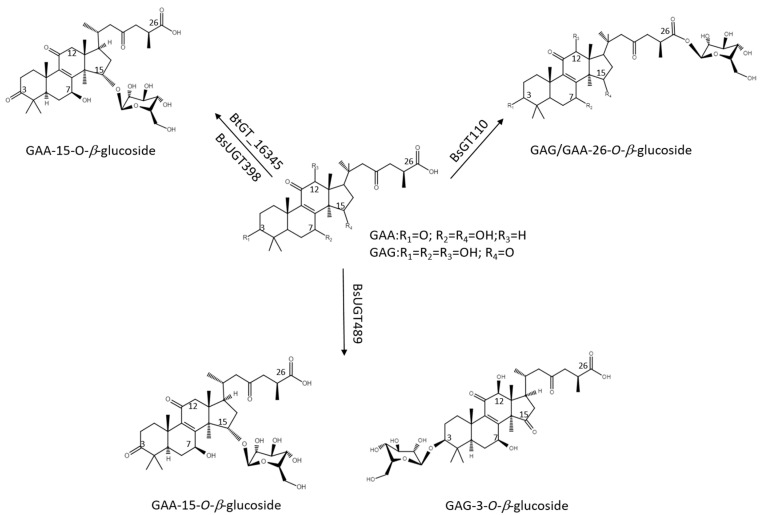
The glycosylation of GAA and GAG by the four *Bacillus* GTs.

The four *Bacillus* GTs have been considered genetically distinct among the known triterpenoid-catalyzed GTs until now (Figure 1). Previous studies revealed that BsUGT398, BsUGT489, and BtGT_16345 have catalytic specificity toward glycosylating the C-15 hydroxyl group of the GAA triterpenoid [16,17]. In addition, BsGT110 was the first GT that glycosylated the C-26 carboxyl group of the GAA triterpenoid. Moreover, BsGT110 has shown optimal activity at an acidic pH of 6, but a loss of most activity at an alkaline pH of 8. This suggests that BsGT110 might catalyze other triterpenoid substrates under acidic conditions [15], and is further confirmed by our results. BsGT110 also glycosylated the carboxyl group of GAG under acidic condition (Figure 3 and Figure 4). In addition, BsUGT489 was found to glycosylate the C-3 hydroxyl group of GAG. Table 1 and Figure 5 summarize the comparative results of the biotransformation of GAA [15,16,17] and GAG with the four *Bacillus* GTs. The main structure of ganoderic acid contains five functional groups that could be modified: the C-3, C-7, C-12, and C-15 hydroxyl groups, and the C-26 carboxyl group. GAA and GAG are ganoderic acid derivatives. The structure of GAA contains the C-7 and C-15 hydroxyl groups, and the C-26 carboxyl group. The structure of GAG contains the C-3, C-7, and C-12 hydroxyl groups and the C-26 carboxyl group. Accordingly, previous results revealed that (1) BsGT110 regioselectively glycosylates the C-26 carboxyl groups of both GAA and GAG; (2) both BtGT_16345 and BsUGT398 prefer to glycosylate the C-15 hydroxyl group of GAA; (3) BsUGT489 glycosylates the C-3 and C-15 hydroxyl groups of GAG and GAA, respectively. In none of these cases did the four *Bacillus* GTs glycosylate the C-7 or C-12 hydroxyl groups of GAA or GAG. We theorize that the enzymatic preferences of these *Bacillus* GTs may enable the formation of new *Ganoderma* triterpenoid saponins in the future.

### 2.4. Determination of Solubility

The glycosylated molecules demonstrated more advantages than the original aglycone molecules. After glycosylation, the aqueous solubility of the glycosylated derivatives was greatly increased [18,19]. Accordingly, the aqueous solubility of GAG and the two GAG saponins are listed in Table 2. These results show that the aqueous solubility of compound (**1**) and compound (**2**) were 54- and 97-fold greater than that of GAG. Previous studies revealed that glycosylation can also improve the oral bioavailability of the original molecules [20,21]. Thus, the pharmaceutical application of both novel GAG saponins might be worthy of future study.

### 2.5. Perspective

The biological reason for the joint involvement of GAG and *Bacillus* GTs in the synthesis of fungi saponins remains unclear. However, most of the prior studies about amphiphilic molecules have sought to determine, in advance, those saponins capable of inducing cell toxicity, and we know from these studies that some saponins possess membrane permeabilizing properties. Moreover, saponins have been shown to cause mortality, lower food intake, weight reduction, and developmental problems in insects [22,23]. Plants contain large amounts of triterpenoid saponins and saponins, in turn, avoid plants infected by insect bites. On the other hand, glycosylation is also a well-known detoxification process during drug metabolism [24]. It has been reported that triterpenoids are toxic to bacteria (possessing anti-bacterial activity) and that the glycosylation of triterpenoids to saponins reduces triterpenoids toxic effects on bacteria. Accordingly, one possible explanation is that GTs help bacteria to survive with the fungi by glycosylating the fungal triterpenoids to detoxed saponins.

## 3. Materials and Methods 

### 3.1. Enzymes and Chemicals

GAG was purchased from Baoji Herbest Bio-Tech (Xi’an, China). UDP-G was obtained from Cayman Chemical (Ann Arbor, MI, USA). The recombinant *Bacillus* GTs, BsGT110 [15], BsUGT398, BsUGT489 [16], and BtGT_16345 [17] were obtained from our previous studies. Organic solvents, including methanol and dimethyl sulfoxide (DMSO), and nylon membrane were bought from Sigma (St. Louis, MO, USA).

### 3.2. Biotransformation

The in vitro biotransformation was conducted using the purified recombinant GTs. The optimal reaction conditions for GAA glycosylation, suggested in previous studies, were used as the reaction conditions for GAG glycosylation [15,16,17]. The reaction mixture (1 mL) contained 25 μg/mL of the tested enzyme, 1 mg/mL of GAG, 10 mM of UDP-G, 10 mM of MgCl_2_, and 50 mM of buffer at different pHs (see below). The reactions were carried out at 30 °C for BsUGT398 and BtGT_16345 for 30 min, and at 40 °C for BsUGT489 and BsGT110 for 30 min. For different enzyme reactions, different buffers were used: phosphate buffer (PB) at pH 6.0 for BsGT110, PB at pH 7.0 for BtGT_16345, and Tris buffer at pH 8.0 for BsUGT398 and BsUGT489. Reactions were ended by adding an equal volume of methanol and the mixture was then analyzed using HPLC.

### 3.3. HPLC Analysis

HPLC was performed using the Agilent^®^ 1100 series HPLC system (Santa Clara, CA, USA) equipped with a gradient pump (Waters 600, Waters, Milford, MA, USA). The stationary phase was a C18 column (Sharpsil H-C18, 5 μm, 4.6 i.d. × 250 mm, Sharpsil, Bei-jing, China) and the mobile phase was 1% acetic acid in water (A) and methanol (B). The elution condition was a linear gradient from 0 min with 40% B to 20 min with 70% B; isocratic from 20 min to 25 min with 70% B; linear gradient from 25 min with 70% B to 28 min with 40% B; and isocratic from 28 min to 35 min with 40% B. The flow rate of the mobile phase was 1 mL/min. The sample volume was 10 μL. Detection intensity was set at 254 nm.

### 3.4. Purification and Identification of the Biotransformation Products

To purify the biotransformation metabolites, the biotransformation reactions using either BsUGT489 or BtGT_16345, described above, were scaled up to 20 mL. Equal volumes of methanol were added to stop the reactions. After filtration through a 0.2 μm nylon membrane, the mixtures were injected into a preparative YoungLin HPLC system (YL9100, YL Instrument, Anyang, Korea). The system was equipped with a preparative C18 reversed-phase column (Inertsil, 10 μm, 20.0 i.d. × 250 mm, ODS 3, GL Sciences, Eindhoven, The Netherlands). The operational conditions for the preparative HPLC were the same as those for HPLC. The elution corresponding to the peak of the product in UPLC was collected, concentrated under vacuum, and then lyophilized. Finally, 14.4 mg of compound (**1**) and 7.8 mg of compound (**2**) were obtained, and the structures of both compounds were confirmed using NMR and mass spectrometry. Mass spectrometry was performed using a Finnigan LCQ Duo mass spectrometer (ThermoQuest Corp., San Jose, CA, USA) with ESI. The ^1^H- and ^13^C-NMR, DEPT, HSQC, HMBC, COSY, and NOESY spectra were recorded using a Bruker AV-700 NMR spectrometer (Bruker Corp., Billerica, MA, USA) at 25 °C. Standard pulse sequences and parameters were used for the NMR analyses and all chemical shifts were reported in parts per million (ppm, δ).

### 3.5. Determination of Solubility

The aqueous solubility of GAG and its glucoside derivatives were examined as follows. One milligram of each compound was vortexed in 0.1 mL of deionized water for 1 h at 25 °C. The mixture was then centrifuged at 10,000× *g* for 30 min and the supernatant was filtered through a 0.2 μm nylon membrane at 25 °C. The filtrate was mixed with an equal volume of methanol and analyzed using HPLC. The concentrations of the tested compounds were determined based on their peaks using calibration curves prepared using HPLC analyses of the authentic samples dissolved in DMSO.

## 4. Conclusions 

Two new and highly aqueous-soluble *Ganoderma* triterpenoid saponins, GAG-3-*o*-*β*-glucoside (**1**) and GAG-26-*o*-*β*-glucoside (**2**), were produced by the biotransformation of GAG using BsUGT489 and BsGT110, respectively. This study revealed that the glycosylation site preferences of the different functional ganoderic acid groups vary among the different *Bacillus* GTs. BsGT110 regioselectively glycosylates the C-26 carboxyl group of GAA and GAG. Both BtGT_16345 and BsUGT398 prefer to glycosylate the C-15 hydroxyl group of GAA. BsUGT489 glycosylates the C-3 and C-15 hydroxyl groups of GAG and GAA, respectively. This study highlights the possible application of *Bacillus* GTs for purposes of forming future *Ganoderma* triterpenoid saponins.

## Figures and Tables

**Table 1:** Glycosylation activities of the four *Bacillus* glycosyltransferases (GTs) toward the different functional groups of GAA and GAG.

Triterpenoid	Functional Groups in Ganoderic Acids				
	C3-OH	C7-OH	C12-OH	C15-OH	C26-COOH
GAA	N.A. ^1^	N.D. ^2^	N.A.	BsUGT489 > BtGT_16345 > BsUGT398 ^3^	BsGT110
GAG	BsUGT489	N.D.	N.D.	N.A.	BsGT110 >> BtBGT_16345

^1^ N.A. means that the functional group is not available in the ganoderic acid. ^2^ N.D. means that the glycosylation of the functional group was not detectable in the reactions of the studied GTs. ^3^ Data obtained from Table 1 of Ref. [17].

**Table 2:** Aqueous solubility of GAG and its derivatives at 25 °C.

Compound	Aqueous Solubility (mg/L) ^1^	Fold ^2^
GAG	18.85 ± 1.36	1
GAG-3-*o*-*β*-glucoside (**1**)	1019.64 ± 9.54	54.0
GAG-26-*o*-*β*-glucoside (**2**)	1829.89 ± 51.21	97.0

^1^ The mean (*n* = 3) are shown, and the standard deviations are represented by error bars. ^2^ The fold of aqueous solubility of GAG glucoside derivatives is expressed as relative to that of GAG, normalized to 1.

## Data Availability

The data presented in this study are available in the article or supplementary material.

## Supplementary Materials

The following materials are available online at https://www.mdpi.com/article/10.3390/ijms22189744/s1.
