# Supplementary material for: Glycosylation of Ganoderic Acid G by Bacillus Glycosyltransferases
Source: Int J Mol Sci. 2021 Sep 9;22(18):9744. doi: 10.3390/ijms22189744 (PMC8468440; doi:10.3390/ijms22189744)
Supplement: Supplementary file 1 [file ijms-22-09744-s001.zip › ijms-1355056-SI.pdf]

Glycosylation of Ganoderic Acid G by *Bacillus* Glycosyltransferases

Jiumn-Yih Wu, [1,+] Hsiou-Yu Ding, [2,+] Tzi-Yuan Wang, [3,+] Yun-Rong Zhang, [4] and Te-Sheng Chang [4,*]

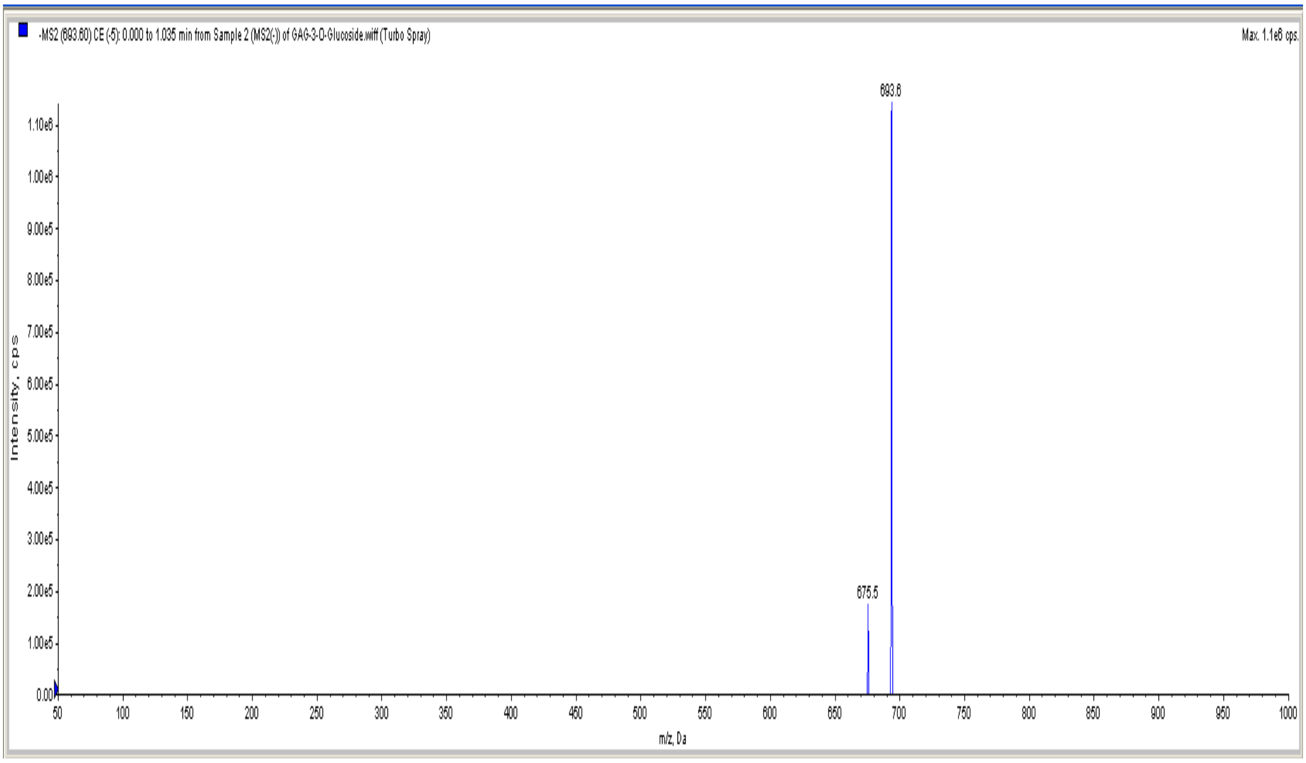

Figure S1. Mass-mass analysis of GAG-3-O-β-glucoside (**1**) at the negative mode. A significant signal at m/z 693.5 showed the corresponding m/z signal of molecular weight 676 of GAG-glucoside (514+180-18) at the negative mode [GAG-G – H]$^{-}$.

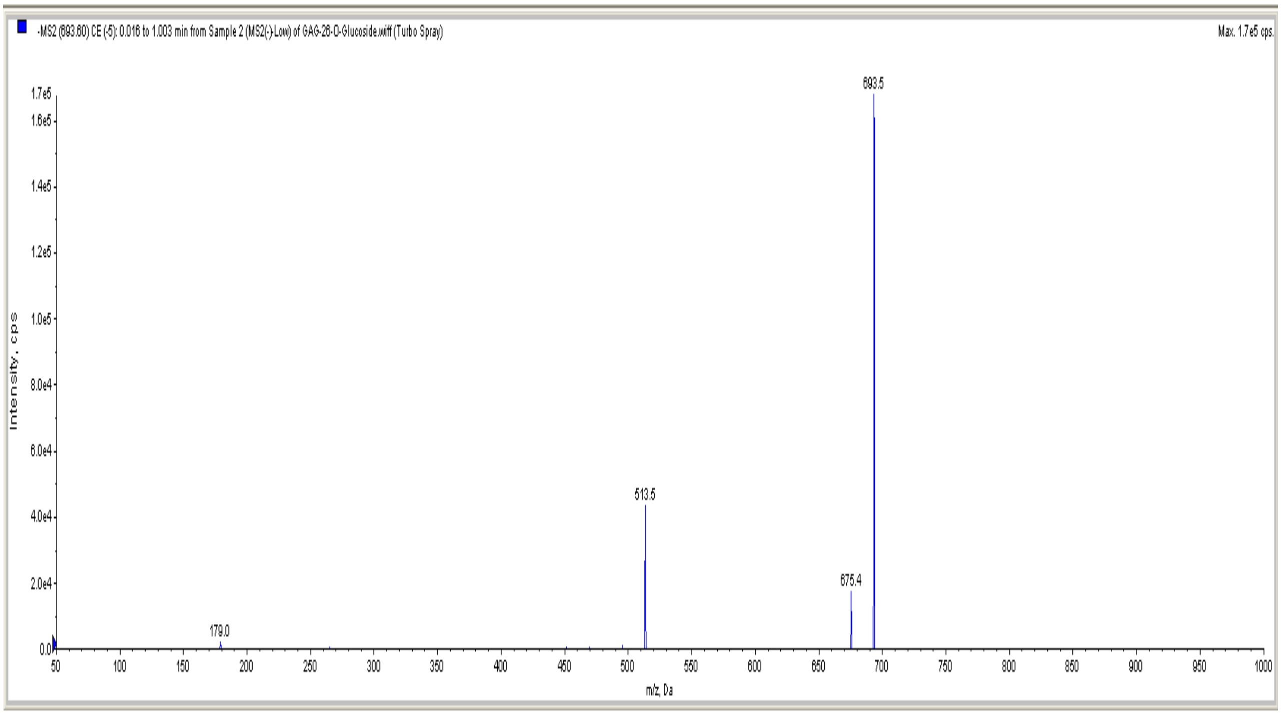

Figure S2. Mass-mass analysis of the m/z 693.5 peak of GAG-26-O-*β*-glucoside (**2**) at the negative mode. Two significant peaks at m/z 675,4 and 513.5 showed the corresponding m/z signals of molecular weights 676 and 514 of GAG glucoside (514+180-18) and GAA (514) at the negative mode [GAG-G – H]$^-$ and [GAG – H]$^-$.

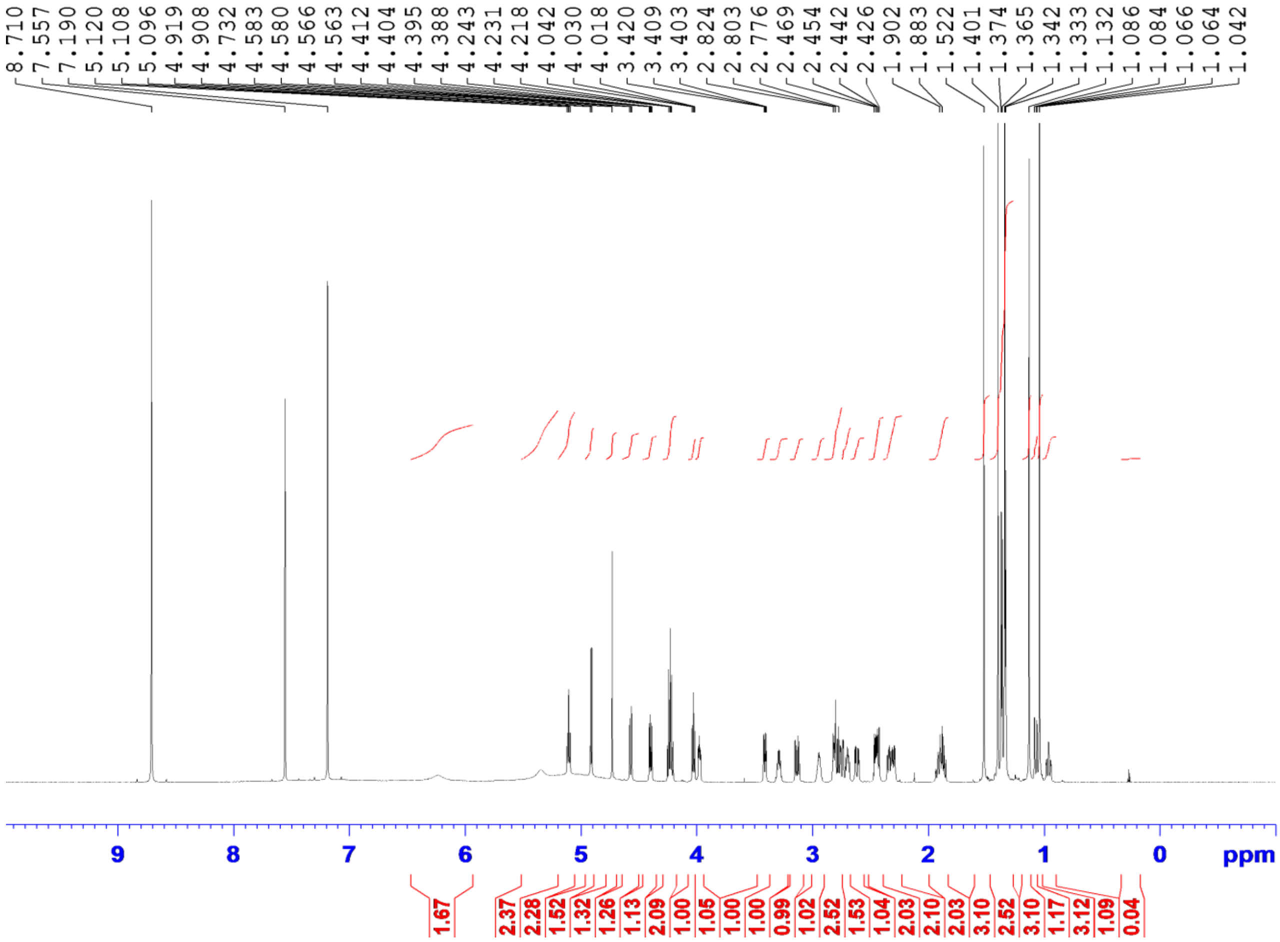

Figure S3. The ^{1}H-NMR (700 MHz) spectrum of GAG-3-O-*β*-glucoside (**1**) in pyridine-d5.

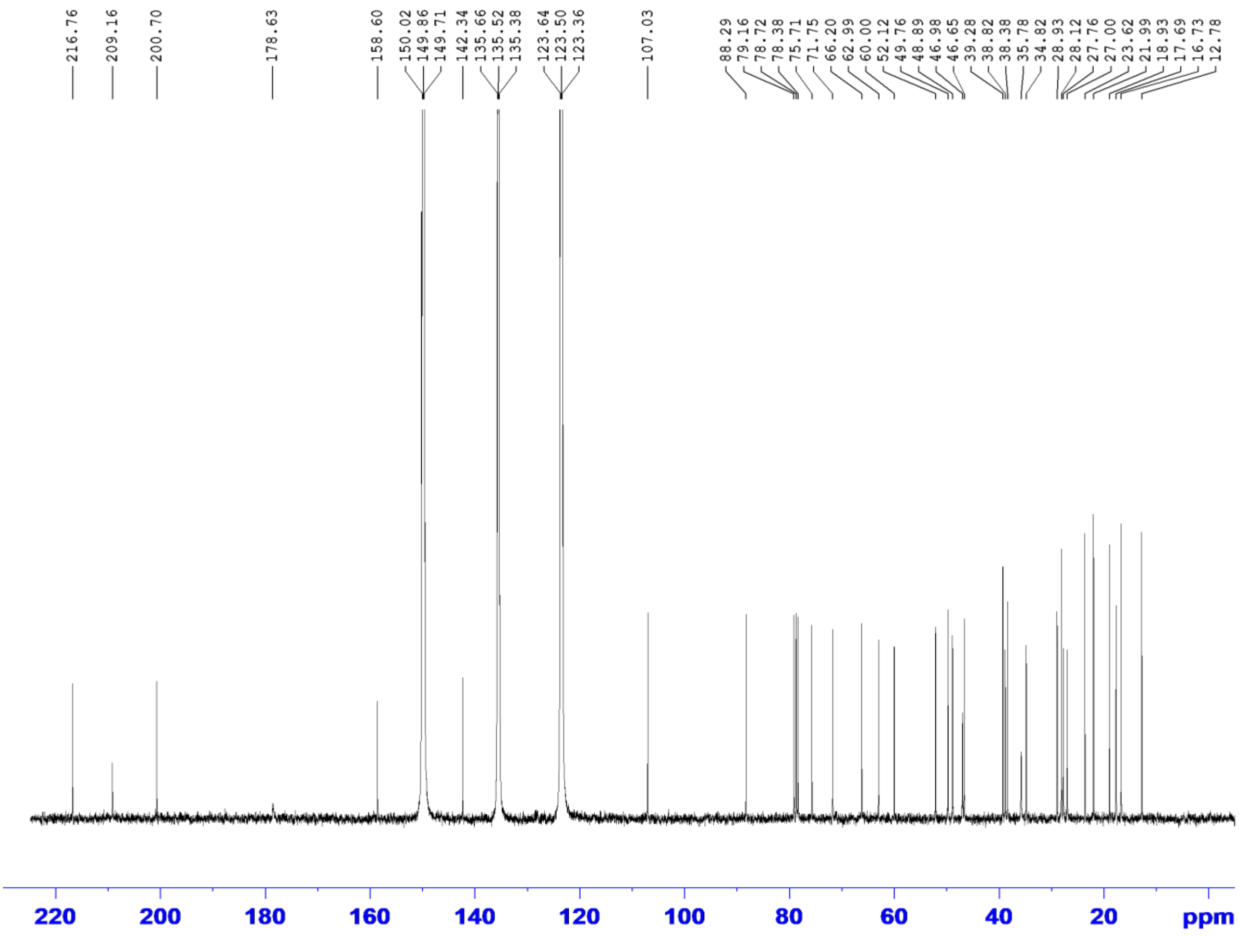

Figure S4. The ^{1}C-NMR (176 MHz) spectrum of GAG-3-O-*β*-glucoside (**1**) in pyridine-d5.

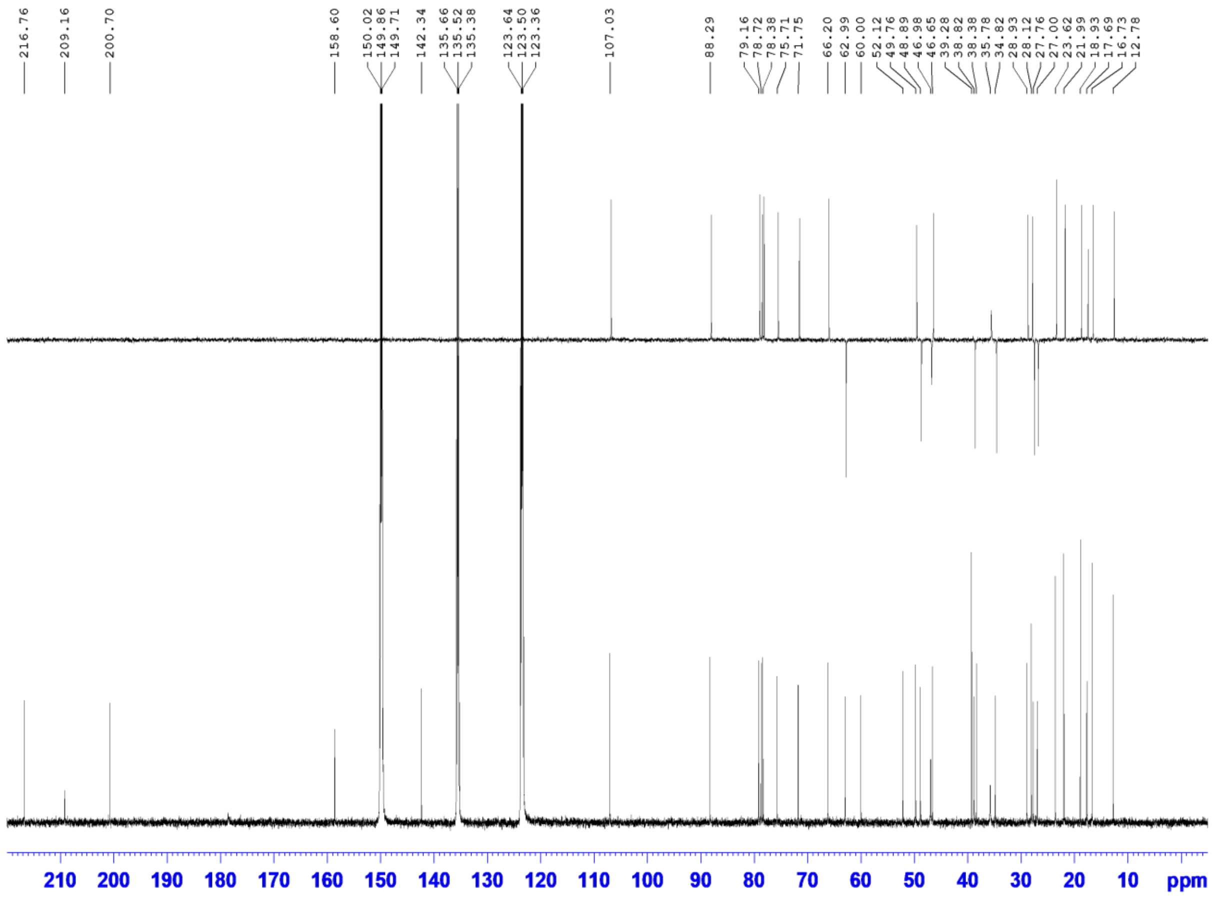

Figure S5. The DEPT-135 (176 MHz) spectrum of GAG-3-O-*β*-glucoside (**1**) in pyridine-d5

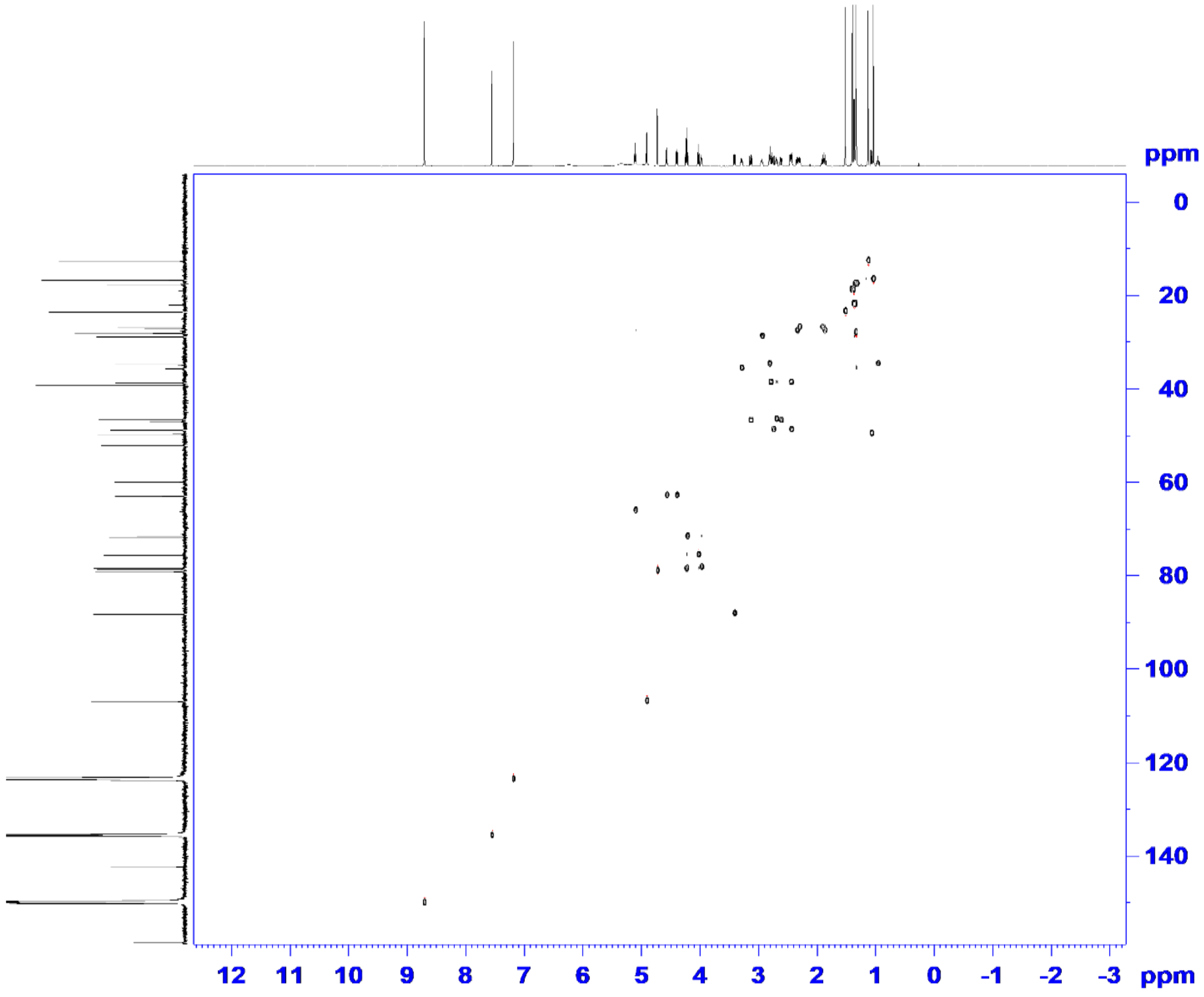

Figure S6. The HSQC (700 MHz) spectrum of GAG-3-O-*β*-glucoside (**1**) in pyridine-d5.

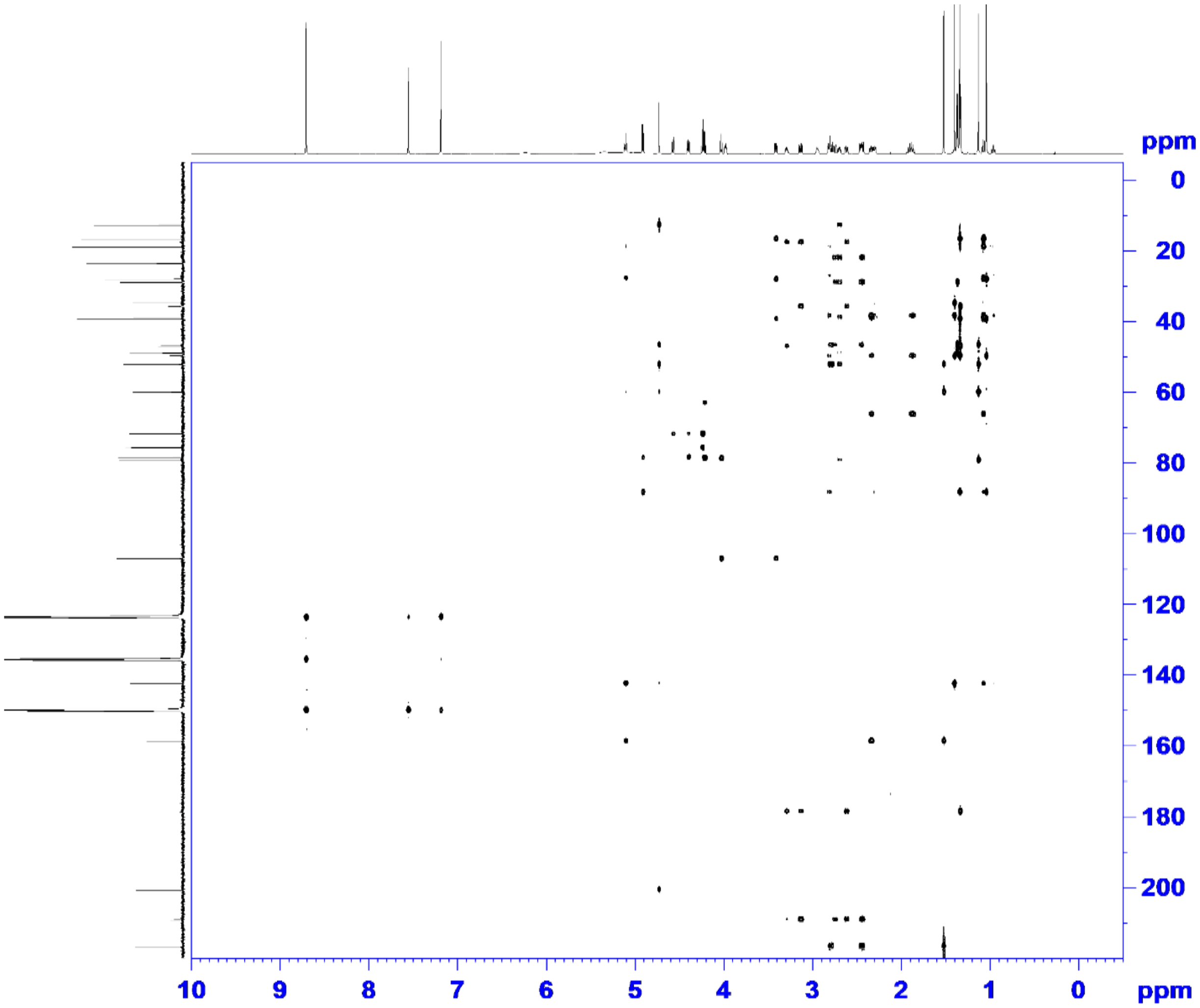

Figure S7. The HMBC (700 MHz) spectrum of GAG-3-O-*β*-glucoside (**1**) in pyridine-d5.

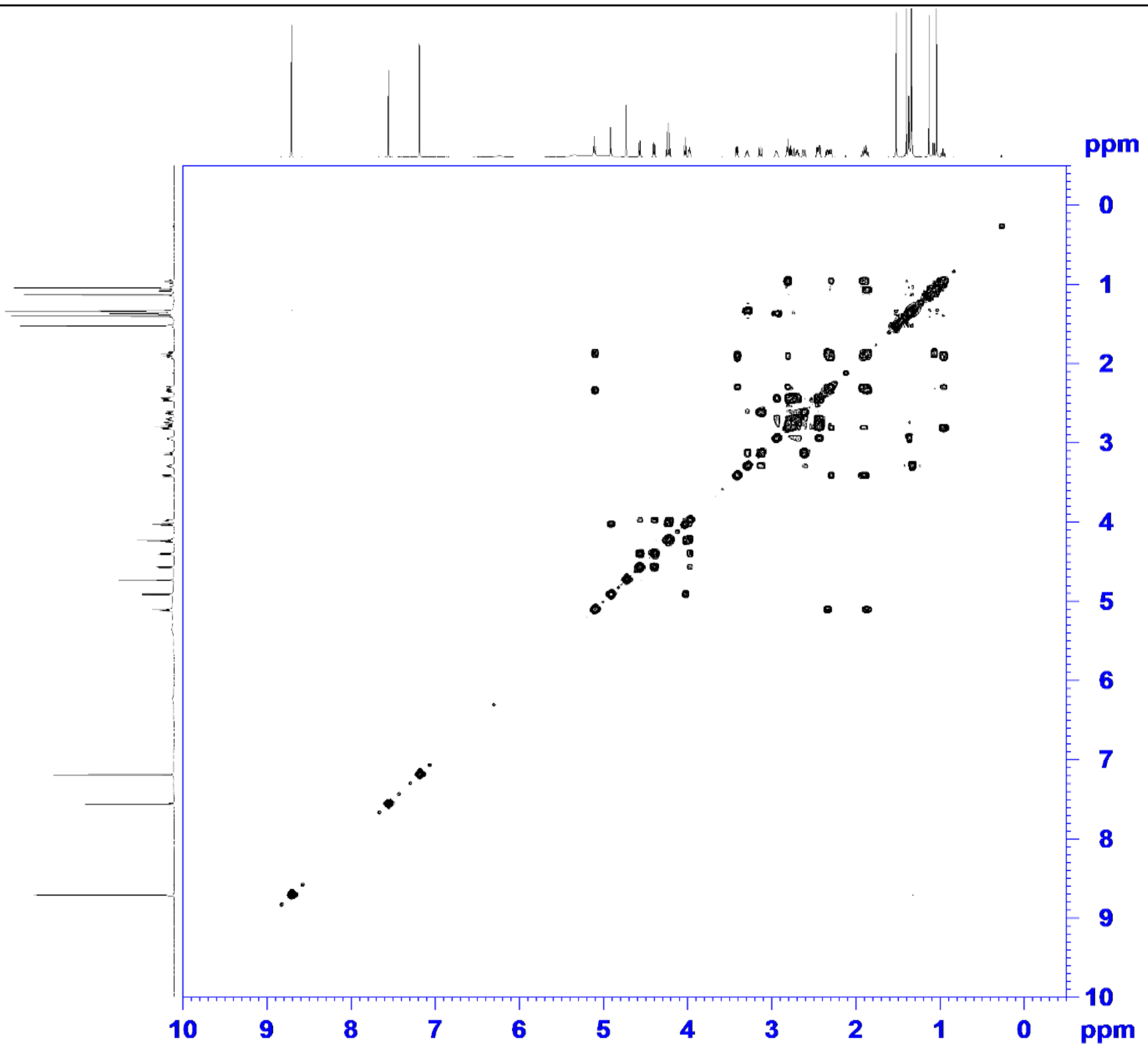

Figure S8. The COSY (700 MHz) spectrum of GAG-3-O-β-glucoside (**1**) in pyridine-d5.

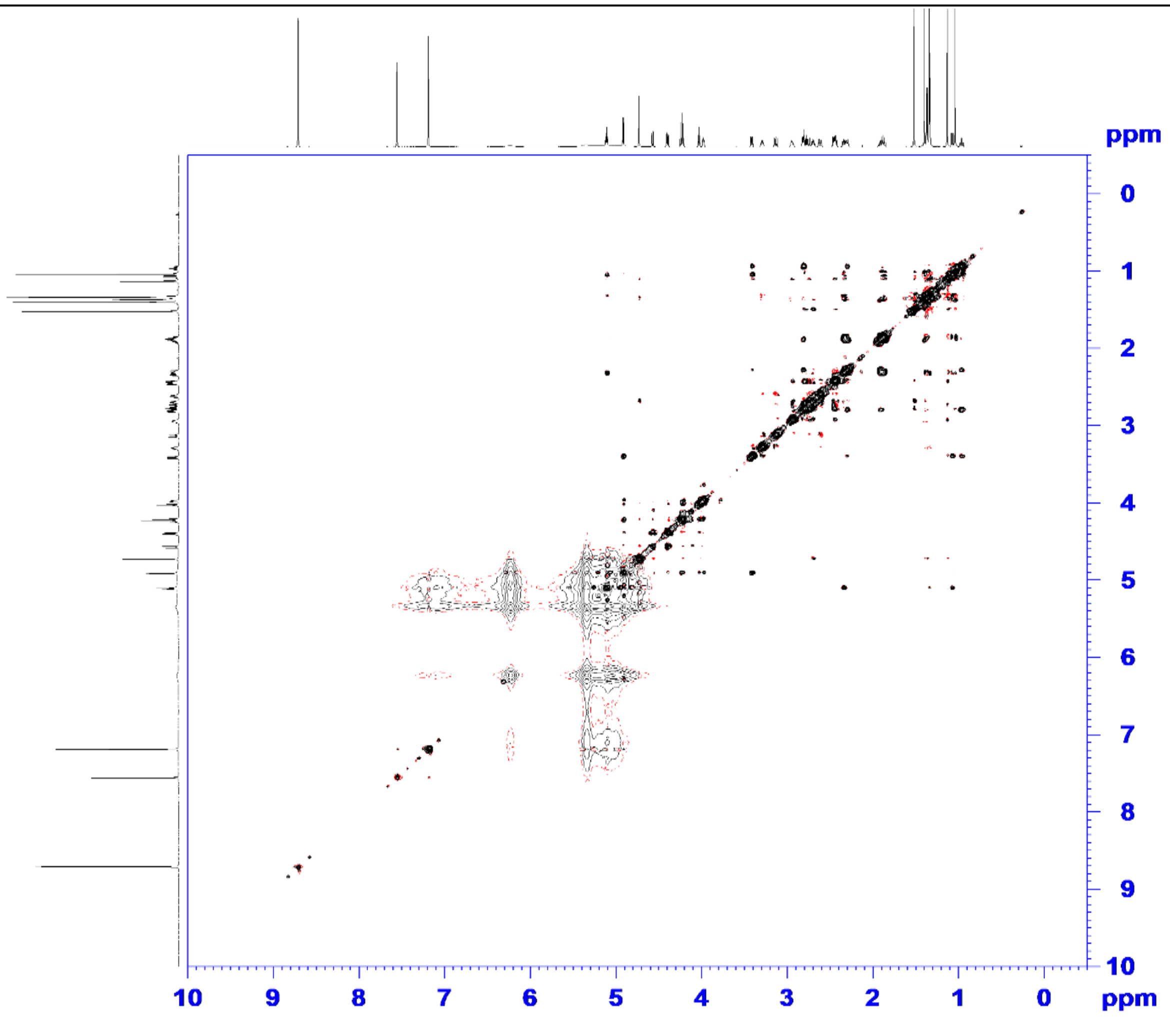

Figure S9. The NOESY (700 MHz) spectrum of GAG-3-O-*β*-glucoside (**1**) in pyridine-d5.

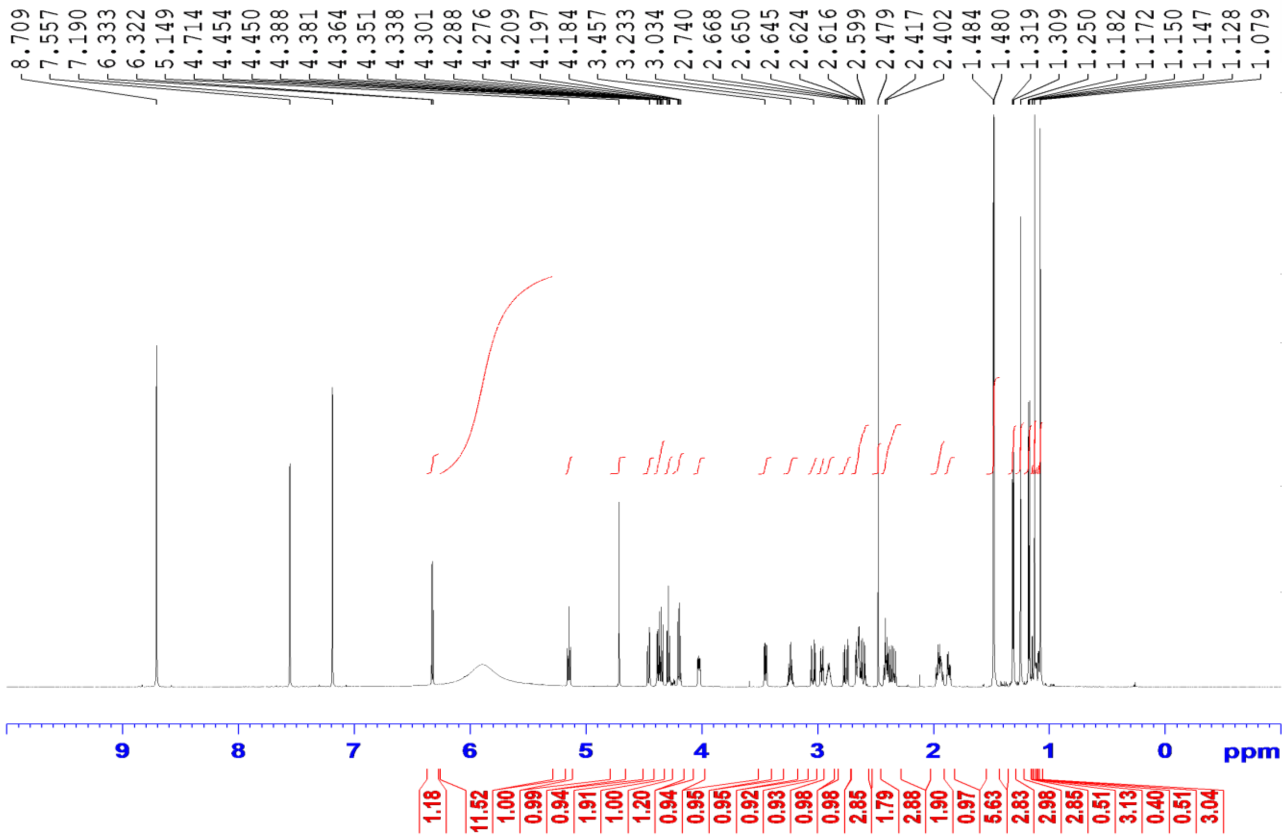

Figure S10. The ^{1}H-NMR (700 MHz) spectrum of GAG-26-O-*β*-glucoside (**2**) in pyridine-d5.

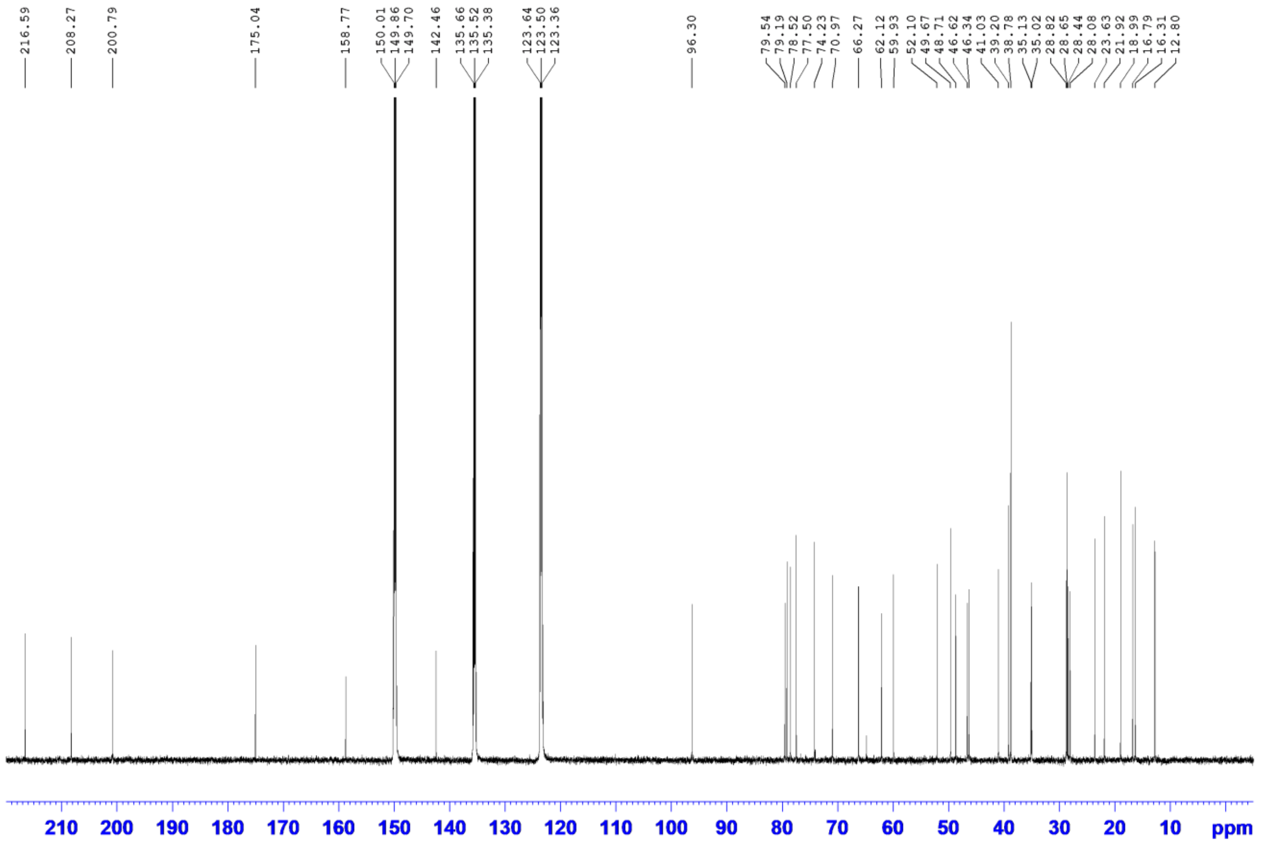

Figure S11. The ^{13}C-NMR (176 MHz) spectrum of GAG-26-O-*β*-glucoside (**2**) in pyridine-d5.

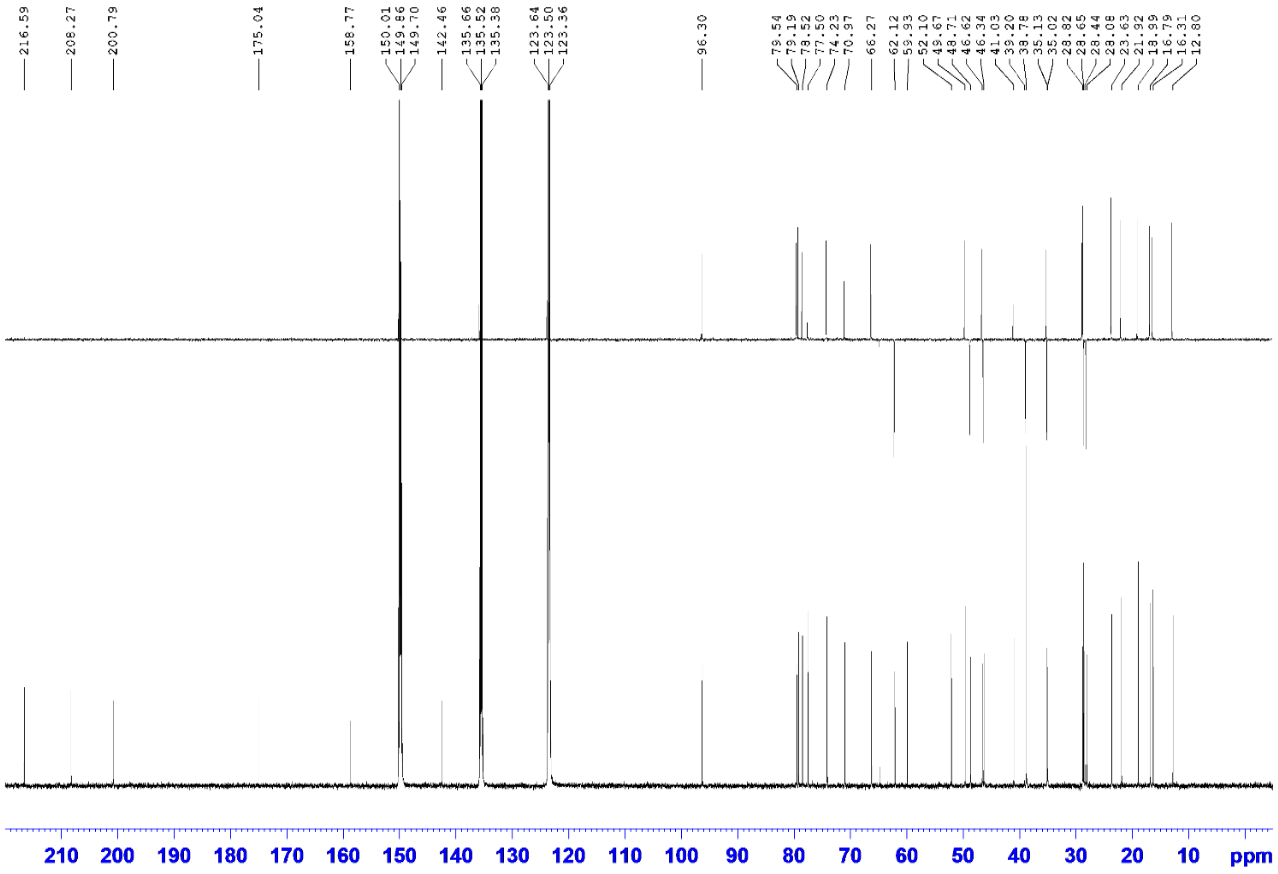

Figure S12. The DEPT-135 (176 MHz) spectrum of GAG-26-O-*β*-glucoside (**2**) in pyridine-d5.

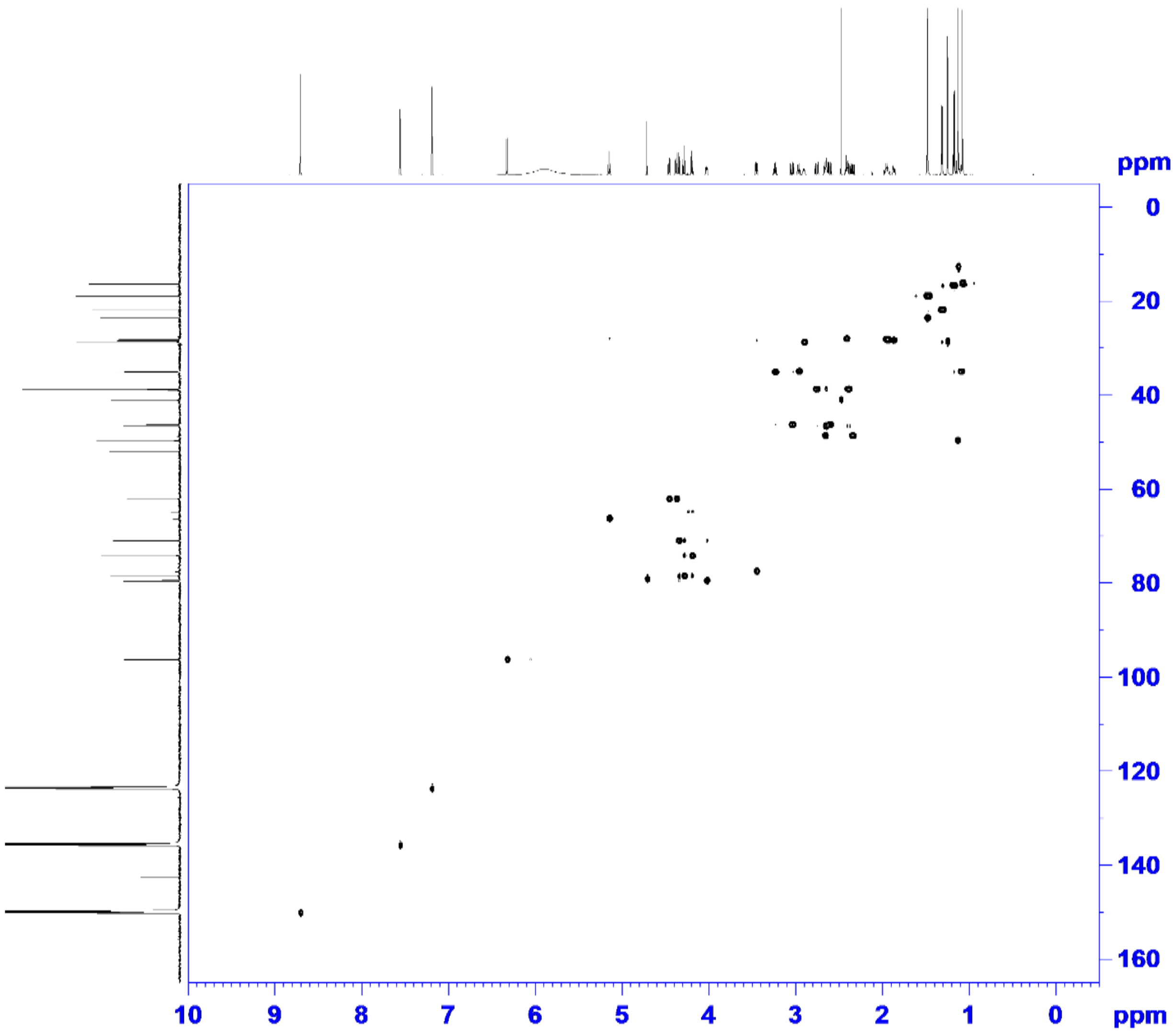

Figure S13. The HSQC (700 MHz) spectrum of GAG-26-O-β-glucoside (**2**) in pyridine-d5.

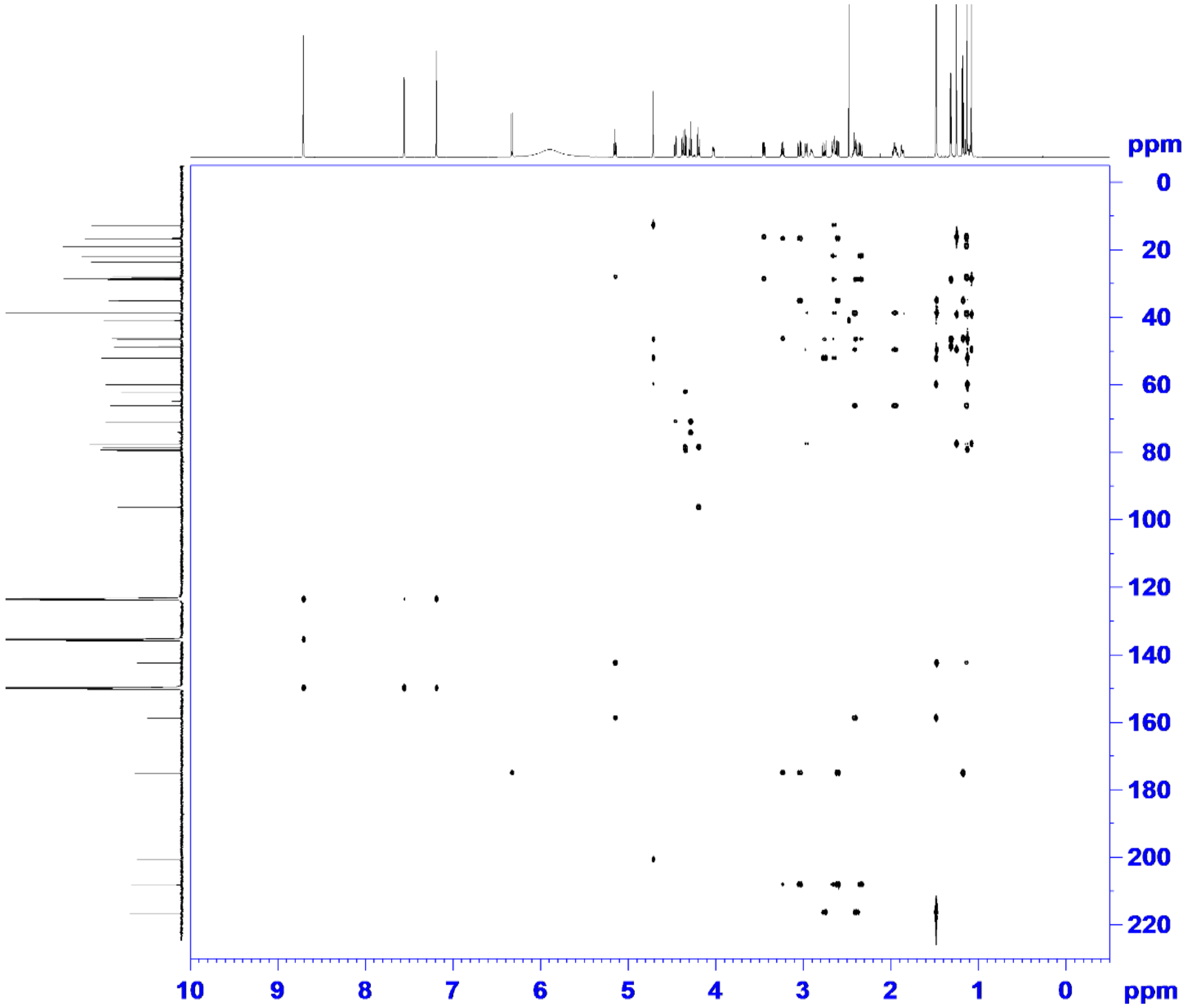

Figure S14. The HMBC (700 MHz) spectrum of GAG-26-O-*β*-glucoside (**2**) in pyridine-d5

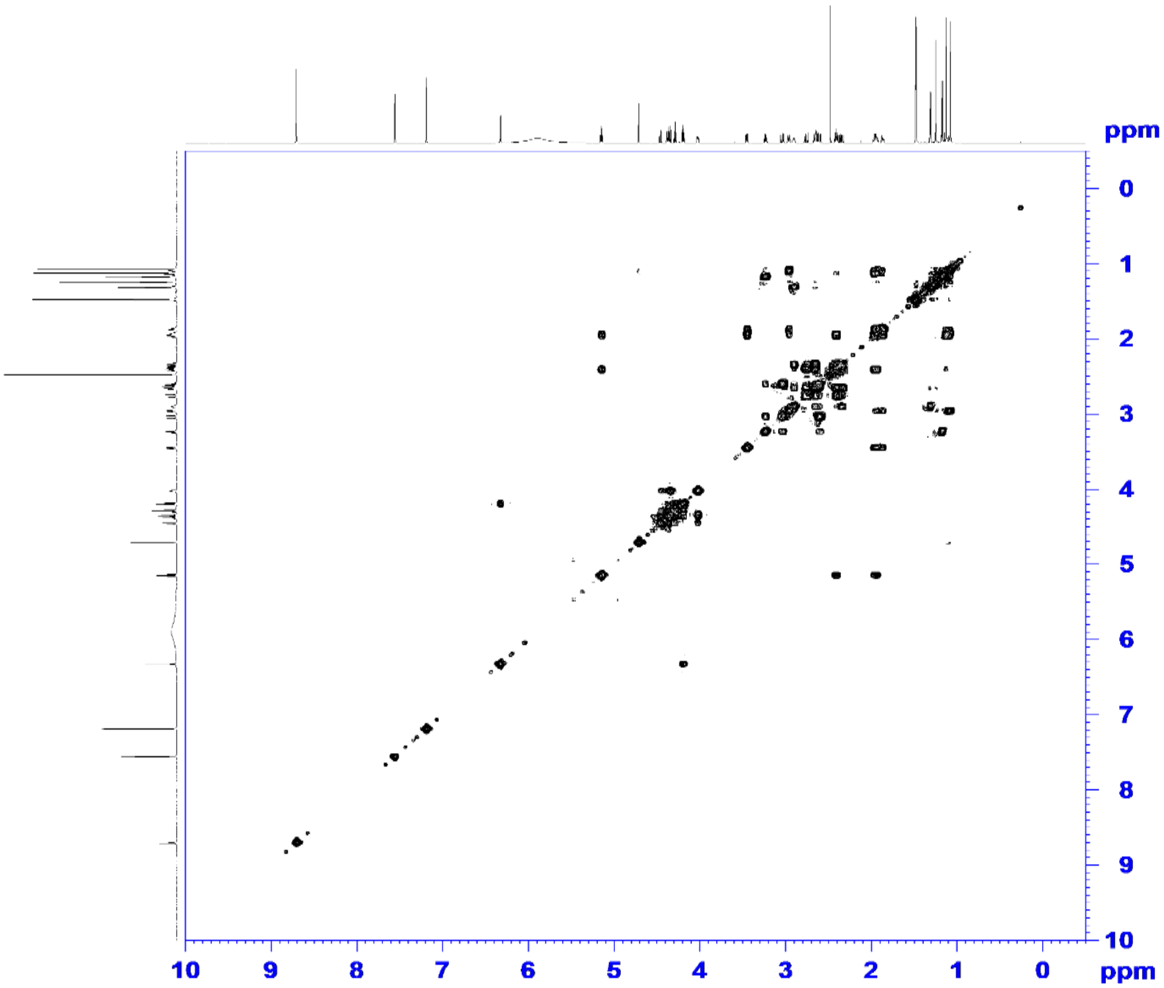

Figure S15. The COSY (700 MHz) spectrum of GAG-26-O-β-glucoside (**2**) in pyridine-d5.

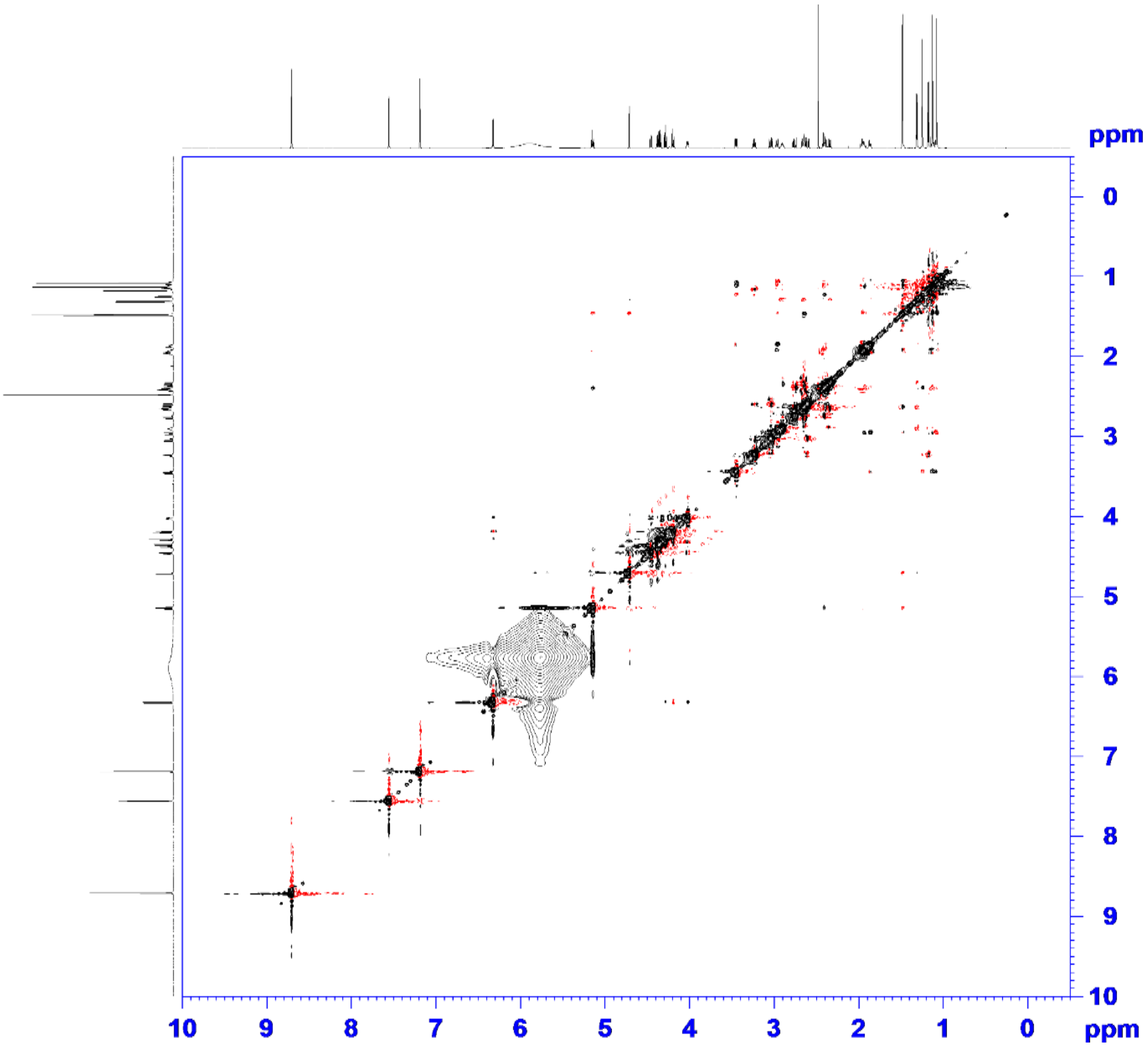

Figure S16. The NOESY (700 MHz) spectrum of GAG-26-O-β-glucoside (**2**) in pyridine-d5
